# Identification of differentially expressed genes in *Monochamus alternatu*s digested with azadirachtin

**DOI:** 10.1038/srep33484

**Published:** 2016-09-15

**Authors:** Tong Lin, Qisi Liu, Jingxiang Chen

**Affiliations:** 1 College of Forestry and Landscape Architecture, South China Agricultural University, Guangzhou, 510642, China

## Abstract

The pine sawyer beetle *Monochamus alternatus* Hope, a major forest insect pest, is the primary vector of the destructive forest pest pine wood nematode, *Bursaphelenchus xylophilus*. Azadirachtin, an active compound of neem, is biologically interesting because it represents a group of important, successful botanical pesticides. We provide insight into the molecular effects of azadirachtin on *M. alternatus* at the transcriptional level to provide clues about possible molecular-level targets and to establish a link between azadirachtin and insect global responses. We found that 920 and 9894 unique genes were significantly up- and down-regulated, respectively. We obtained expression patterns of the differentially expressed genes (DEGs), identifying 4247, 3488 and 7613 sequences that involved cellular components, molecular functions and biological processes, respectively, and showed that the DEGs were distributed among 50 Gene Ontology categories. The Encyclopedia of Genes and Genomes pathway enrichment analysis indicated that the DEGs were enriched in 50 pathways. Detailed gene profile knowledge of the interaction of azadirachtin with *M. alternatus* should facilitate the development of more effective azadirachtin-based products against *M. alternatus* and other target Coleoptera. These results further enhance the value of azadirachtin as a potential insecticide of biological origin, as well as for other biological applications.

The pine sawyer beetle *Monochamus alternatus* Hope (Coleoptera: Cerambycidae) is the primary vector of the destructive forest pest pine wood nematode *Bursaphelenchus xylophilus* (Steiner et Buhrer) Nickle (Aphelenchida: Parasitaphelenchidae) and also causes serious damage to several pine species^1^. Chemical insecticides have been used to control insects for many years. However, pesticide resistance, environmental pollution, and inaccessible larvae within the wood of trunks and branches have largely prevented successful longhorned beetle control in tree plantations^2^,^3^,^4^.

It is vital that ecosystem disruption through the misuse of chemical pesticides be avoided; therefore, a safe, efficient and eco-friendly pest control strategy is required^5^. One alternative is the use of a botanical insecticide, azadirachtin, a member of the tetranortriterpenoid (limonoid) family that is obtained from *Azadirachta indica* (Neem, Meliaceae). It is one of the most biologically active natural inhibitors of insect growth and development. Since it was first found to be a feeding deterrent to the swarming desert locust, *Schistocerca gregaria*, in India, it has been shown to be effective in more than 500 insect species^6^,^7^. Recently, azadirachtin has been assessed in significant biopesticides, has increasingly been used for integrated pest management (IPM) programs^8^, and has been widely used to control a variety of insect pests, such as Coleoptera, Hemiptera and Lepidoptera.

As a representative natural pesticide, however, azadirachtin has still not achieved a prominent place among pesticides, and in many countries, it is not yet licensed for use for many reasons, with unclear mechanisms of action being one of the most striking barriers to licensure^9^. Although molecular techniques have been used to determine its mode of action, and the expression of some genes affected by azadirachtin have been analyzed using real-time RT-PCR analysis^10^, the mode of action of azadirachtin is poorly understood at the cellular and molecular levels^9^.

In spite of the numerous publications on azadirachtin-insect relations, no research exists on *M. alternatus,* which is a major forest insect pest to many pines, especially to *Pinus massoniana*. In the present study, we provide insight into the molecular effects of azadirachtin on *M. alternatus* at the transcriptional level using an Ion Proton next-generation sequencing approach to provide possible molecular-level targets and to establish a link between azadirachtin and global insect responses. The expected result was a more detailed understanding of the molecular mechanisms of azadirachtin in insect toxicology and immunity.

## Results

### Global gene expression after azadirachtin treatment

**Figure 1:**
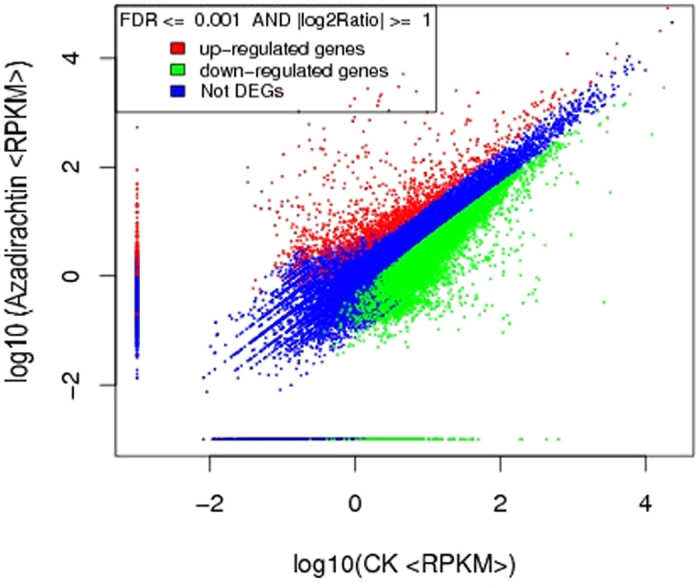
Comparison of the gene expression levels between the CK and azadirachtin-treated *M. alternatus*. To compare gene expression levels between the two libraries, each library was normalized to 1 million tags. The x axis represents Log10 of the reads per kb per million (RPKM) of the control sample, and the y axis indicates Log10 of the RPKM of the treated sample. The expression level of each gene is included in the volcano plot. The red dots represent transcripts that are more prevalent in the azadirachtin-treated library, the green dots show those present at a lower frequency in the azadirachtin-treated library, and the blue dots indicate transcripts that did not change significantly. The parameters “FDR <0.001” and “absolute value of log2 (Treat/Control) ≥1” were used as thresholds to judge significance differences in gene expression.

The differentially expressed genes (DEGs) were analyzed by pairwise comparisons of control and azadirachtin-treated *M. alternatus*. We found that 920 and 9894 unique genes were significantly up- and down-regulated, respectively. The unigenes detected with at least two-fold differences in the two libraries are shown in Fig. 1 (FDR < 0.001). The red and green dots represent transcripts that are found in higher and lower abundance by more than two-fold, respectively. The blue dots represent transcripts that differed by less than two-fold between the two libraries, which were arbitrarily designated as “no difference in expression”.

### Gene ontology functional classification of DEGs

Gene ontology (GO) is an international standardized gene functional classification system that offers a dynamic, updated controlled vocabulary and a strictly defined concept to comprehensively describe properties of genes and their products in any organism. GO covers three domains: cellular component, molecular function and biological process. The basic unit of GO is a GO-term. Every GO-term belongs to a type of ontology.

**Figure 2:**
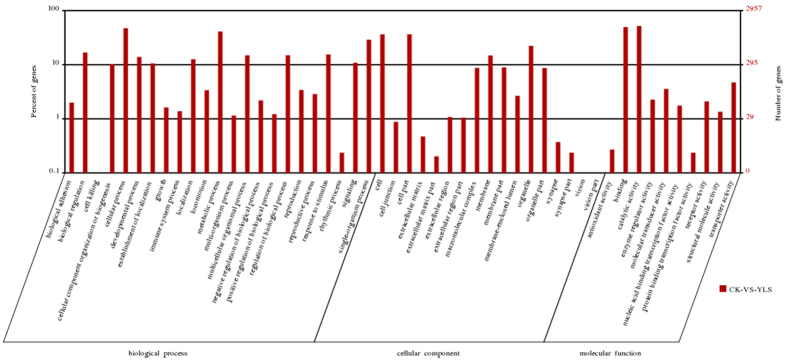
Classification of DEG gene ontology. The DEG functions were described using GO terms.

We obtained the expression patterns of the DEGs annotated to their given GO terms. Annotation of non-redundant sequences using the GO database yielded better results, identifying 4247 sequences as cellular components, 3488 sequences involved in molecular functions and 7613 sequences involved in biological processes, and showed that the genes were distributed among 50 categories, including developmental process (411 genes), growth (48 genes), immune system process (41 genes), reproduction (101 genes), response to stimulus (458 genes) and enzyme regulator activity (67 genes). The most abundant GO biological process categories were cellular process (1395 genes) and metabolic process (1219 genes). Similarly, the most abundant GO molecular function categories were catalytic activity (1540 genes) and binding (1467 genes), and the most abundant GO cellular components were cell (1079 genes) and cell part (1079 genes) (Fig. 2). The highest-rated GO terms for the three GO categories are shown in Table 1.

### KEGG pathway enrichment analysis of DEGs

It is believed that a molecular biological approach based on understanding insect molecules and their associated biochemical pathways will be helpful in the design and development of novel and effective control agents. The Encyclopedia of Genes and Genomes (KEGG) pathway enrichment analysis of DEGs showed that DEGs were enriched in 50 pathways with *P* values less than 0.01.

**Figure 3:**
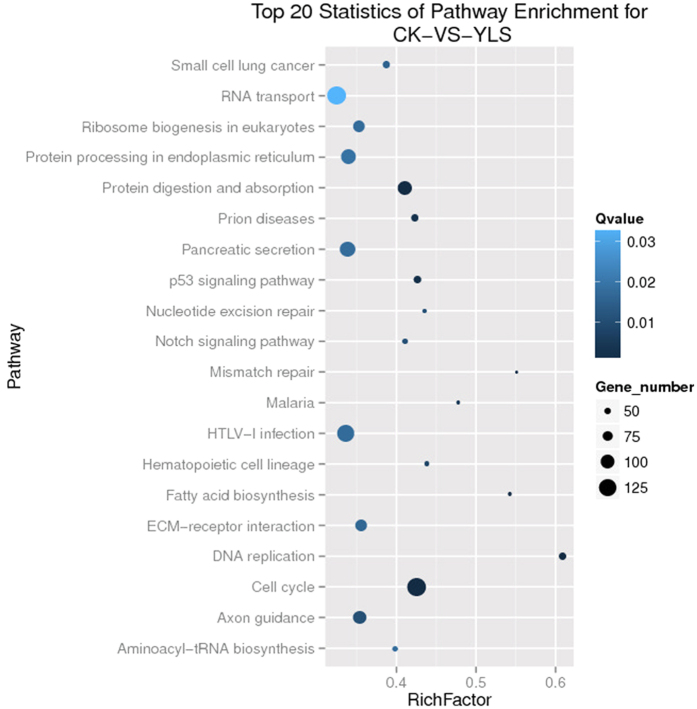
Top 20 enriched pathways for azadirachtin treated with *M. alternatu*s vs. control.

In addition, we generated a scatter plot of the KEGG enrichment results (Fig. 3). RichFactor is the ratio of the differentially expressed gene numbers annotated in this pathway term to all gene numbers annotated in this pathway term. A greater richFactor value means greater intensiveness. The Qvalue is the corrected p-value ranging from 0 to 1, and a lower value represents greater intensiveness. We only displayed the top 20 enriched pathway terms in the figure.

### Confirmation of gene expression results by RT-qPCR

**Figure 4:**
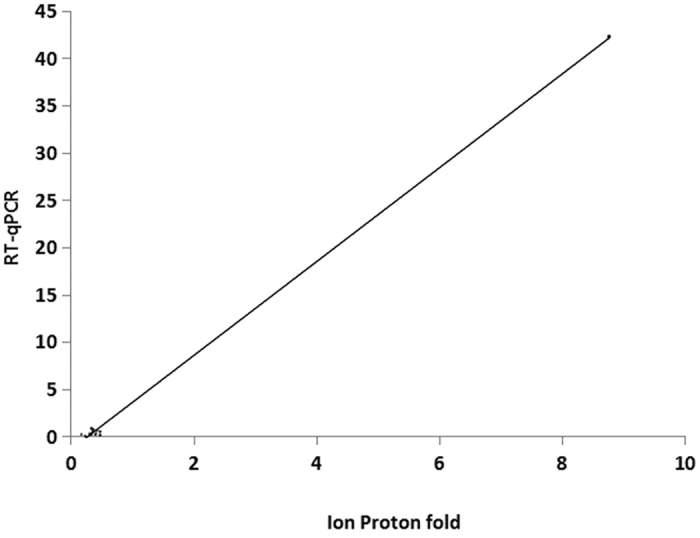
Plotted regression of RT-qPCR validation analysis.

To validate the Ion Proton expression profiles, we randomly selected 13 genes that showed differential expression for further analysis by RT-qPCR. Actin, which was constitutively expressed in our previous work, was chosen as a reference gene for data normalization. The graph in Fig. 4 demonstrates a regression in which the x-axis shows the Ion Proton fold change and the y-axis shows the RT-qPCR results. The regression equation is y = 4.9684x − 1.4218. The correlation coefficient and *P*-value for the regression are 0.9985 and 0, respectively.

## Discussion

Azadirachtin, a natural product that has insecticidal properties and the potential for systemic control of wood-boring and foliar pests^11^, has powerful antifeedant, antifertility, and growth-regulating properties in insects^12^, causing inhibition of insect growth, malformation, and mortality^13^.

Despite the extensive application of azadirachtin in pest control, its mode of action remains controversial. Current evidence suggests that azadirachtin is highly reactive with various cellular molecules in the cytoplasm and nucleus. Azadirachtin may also alter the activity of specific genes and proteins^14^. Therefore, we expected that azadirachtin might regulate various genes at the transcriptional level.

Molecular techniques have been used to determine azadirachtin’s mode of action and molecular interactions at cellular and molecular levels. Lynn *et al.*^10^ evaluated the effects of azadirachtin on the transcription rate of nine selected genes in the Indian meal moth, *Plodia interpunctella*^10^. However, they failed to note the key targets of azadirachtin that could link cell cycle arrest, induction of apoptosis and inhibition of proliferation into a connected framework.

To learn more about the effect of azadirachtin on insects, we created a differentially expressed gene library of *M. alternatus* by azadirachtin ingestion. This study provides a first step toward understanding the profile of azadirachtin targets in *M. alternatus*. The identification of differential targets is a prerequisite for following and understanding the biochemical mechanism by which azadirachtin exerts its effects.

Heat shock protein 70 (HSP70) is a highly conserved family of universal cytosolic chaperones and plays an essential role in protein metabolism under normal and stressed conditions^15^. This protein is also an essential factor in the activation of many signal proteins, such as steroid hormone receptors, the cell cycle kinase Cdk4, and serine/threonine kinases^16^ and is important in immune defense reactions^17^. Each *hsp* gene involved in the GO term of response to a stimulus can respond differentially to various extracellular stimuli, such as temperature extremes, desiccation, toxic substances, and pathogens^18^,^19^. Our results showed that *hsp70* (GO: 0050896) was upregulated 2.08-fold by azadirachtin ingestion. This increased expression of *hsp70* may be associated with the inhibition of cellular growth and proliferation in the larvae that ingested azadirachtin. In addition, increased expression of HSP70, a molecular chaperone, would be triggered by the accumulation of denatured proteins, such that the host cell could prevent the formation of additional denatured proteins^20^. It is possible that upregulation of *hsp70* may increase the ability of proteins to protect themselves from damage during synthesis, folding, assembly, and localization of proteins in cells.

Azadirachtin modulates ecdysteroid hormone action. This natural compound inhibits the release of prothoracicotropic hormone (PTTH) from the corpous cardiacum, a neurohemal organ that lies posterior to the brain^21^. Our results showed that *M. alternatus* larvae that consumed azadirachtin downregulated expression of the nuclear ecdysteroid receptor gene *ultraspiracle (USP*, GO: 0009755//hormone-mediated signaling pathway), a heterodimer of the ecdysone receptor (EcR) by 2.67-fold, indicating a potential interaction between azadirachtin and the ecdysteroid hormonal system. If azadirachtin directly or indirectly influences the expression of this receptor, it could cause a major disruption to insect growth and development. It is possible that the decreased mRNA level of the hormone receptor gene may constrain the action of ecdysteroid hormone, and the growth and molting rates could be significantly inhibited by azadirachtin. Huang *et al.*^22^ also reported that EcR is one of the proteins that is downregulated by the ingestion of azadirachtin in *Spodoptera litura* pupae^22^. Thus, it is likely that transcriptional downregulation of ecdysteroid receptor genes by azadirachtin is associated with EcR protein levels or with further hormonal regulation of development. Early work has shown that treatment of insects with azadirachtin frequently elicits a delay or a permanent block of molting due to reduced ecdysteroid titer^23^.

Among three immune-related genes of *M. alternatus* larvae, *prophenoloxidase*, which is related with metabolic process (GO: 0008152) and oxidoreductase activity (GO: 0016716), was downregulated 2.28-fold by azadirachtin, but *β-1, 3-glucan recognition protein* and *hemolin* levels were unchanged. Thus, genes associated with immune and detoxification systems were selectively modulated at the transcription level by azadirachtin ingestion^10^.

Our results indicated that azadirachtin generates its neurotoxicity by inhibiting calcium channels (GO: 0006816: calcium ion transport) by 5.28-fold, which decreases the excitability of the *M alternatus* central nervous system. The results support the previous study of Qiao *et al.* who suggested a decrease in calcium channel activity in *Drosophila melanogaster*^24^. The neurotoxicity of azadirachtin on the modulation of calcium channels suggested that the nervous system is also a target of azadirachtin. However, a more specific mode of action for azadirachtin in the neurological circuit and the molecular mechanism of the action require further investigation. These results suggested that azadirachtin might have multiple targets^24^. In contrast, a previous study claimed that azadirachtin has an inhibitory effect on potassium channels in cultured dorsal root ganglion^25^. Unexpectedly, we did not detect any change in the expression of potassium channels in *M.alternatus.*

The azadirachtin target for inhibition of cell division is thought to be cytoskeletal proteins involved in cell meiosis and mitosis via blockade of cell division after DNA synthesis at the stage when the chromosomes separate and cytoplasmic division occurs^26^. In our study, *Filamin-C-like* gene, which is involved in cytoskeletal protein binding (GO: 0008092), was downregulated 3.56-fold, which is consistent with the results of Billker, who noted that azadirachtin has been shown to prevent cytoskeletal assembly/function in the malarial parasite, *Plasmodium berghei*^27^. Azadirachtin prevents the orientation of microtubules into more complex structures, such as mitotic spindles and axonemes. As binding of azadirachtin to axonemes causes disruption of the microtubule cytoskeleton, one of the azadirachtin targets is thought to be cellular cytoskeletal elements, especially tubulin^28^. Our results support this hypothesis as the *tubulin* gene (GO: 0043623: cellular protein complex assembly; GO: 0007017: microtubule-based process) was downregulated 5.13-fold.

Alkaline phosphatase (ALP, E.C.3.1.3.1) and acid phosphatase (ACP, E.C.3.1.3.2) are hydrolytic enzymes that hydrolyze phosphomonoesters under acid or alkaline conditions, respectively. Any impairment in their activity will affect insect gut physiology. We found that the genes encoding for ALP, ACP and lactate dehydrogenase (LDH) were downregulated 2.62-, 1.32- and 8.76-fold, respectively. The effect of azadirachtin insect metabolism observed in the present study can be observed in *Spodoptera litura* Fab., in which similar effects were suggested by a decrease in enzyme activity^29^. Changes in ALP, ACP, and LDH activities after treatment with azadirachtin reflected not only the mode of action but also the physiological situation of insect mid-gut. The direct effect of azadirachtin on enzyme regulation suggests reduced phosphorous liberation for energy metabolism, decreased rate of metabolism, as well as a decreased rate of metabolite transport^29^. It is evident that exposure to *M. alternatus* in the larval diet has significant effects on the activity of several enzymes. Decreases in enzymatic activity are further indications of disturbances in the general metabolism of azadirachtin-treated *M. alternatus.* However, we do not have experimental results to support this. Still, azadirachtin has been shown to be the most active compound in terms of its effects on insect physiology^29^.

In Fig. 4, there are actually 13 spots along the regression line, which is corresponding to 13 verified genes. However, because the fold change of one gene is much greater than that of other 12 genes, the spots are not evenly distributed. Most of spots (12 genes) are crowded together along the lower part of the regression line.

In conclusion, we introduced differentially expressed genes in surviving *M. alternatus* larvae by digestion with azadirachtin and found that multiple molecular targets were significantly enriched in 18 GO categories (with P-values less than 0.05) and 50 KEGG pathways (with P-values less than 0.01). Detailed gene profile knowledge of azadirachtin interaction with *M. alternatus* should facilitate the development of more effective azadirachtin-based products against this devastating pest and other target Coleoptera. To the best of our knowledge, this is the first demonstration of gene expression profiles of insects treated with azadirachtin at the transcriptional level. These results further enhance the value of azadirachtin as a potential insecticide of biological origin, as well as for other biological applications.

## Methods

### Insect Culture and Treatment

*M. alternatus* larvae were originally obtained from dead, infested *P. massoniana* trees. The larvae were reared on artificial diets^30^ in complete darkness at room temperature at 55% relative humidity (RH) until they reached the third instar larval stage.

Azadirachtin powder (purity: 96%) was provided by Xing Zhongcheng Science and Technology Co. Ltd, Wuhan, China. The powder was dissolved in pure acetone, and the solutions were added to the artificial diet. The solutions were thoroughly mixed to distribute the azadirachtin evenly. Acetone was allowed to escape in the aerator, which ensured that the final concentration of azadirachtin was 10 ppm in the feed (We carried out preliminary experiment before azadirachtin treatment according to the reference of Lynn^31^, and found that 10 ppm of azadirachtin in the feed was sublethal for *M. alternatus*.). Larvae used for the experiments were at the start of the third instar, and only those of similar size were used. The larvae were starved for 2–3 h and transferred into clear petri dishes (9 cm in diameter) containing the artificial diet with or without azadirachtin. Each experiment used 30 individuals and was replicated three times.

### RNA extraction

Total RNA was extracted from whole bodies of the third instar larvae 12 h after treatment with azadirachtin using TRIzol reagent (Life Technologies, Carlsbad, CA, US) following the manufacturer’s instructions. The quality and concentration of the RNA samples were examined by agarose gel electrophoresis and spectrophotometer analysis. High-quality total RNA was further purified using an RNeasy mini kit (Qiagen, GmBH, Germany) and an RNase-Free Dnase set (Qiagen, GmBH, Germany).

### Construction of the cDNA library and sequencing by Ion Proton

Total RNA samples were first treated with Dnase I to degrade any possible DNA contamination. After DNaseI digestion, they were purified with RNAClean SPRI beads (Beckman Coulter Genomics).The Agencourt RNAClean XP system utilizes Agencourt’s solid-phase paramagnetic bead technology for high-throughput purification of RNA. It utilizes an optimized buffer to selectively bind RNA or cDNA to paramagnetic beads. Excess oligonucleotides, nucleotides, salts, and enzymes can be removed using a simple washing procedure. Next, the mRNA was enriched using oligo (dT) magnetic beads. The mRNA was then mixed with the fragmentation buffer and fragmented into short fragments (approximately 200 bp). Then, the first strand of cDNA was synthesized using random hexamer-primed reverse transcription. Buffer, dNTPs, Rnase H and DNA polymerase I were added to synthesize the second strand. The double strand cDNA was purified with magnetic beads. End reparation was then performed. After the previous step, adaptors were ligated to the ends of these fragments. Next, ligation products were selected by size and purified on TAE-agarose gel. Finally, the fragments were enriched by PCR amplification, then purified by magnetic beads and dissolved in the appropriate amount of Epstein-Barr solution. An Agilent 2100 Bioanaylzer was used to qualify and quantify the sample library. The library products were sequenced via the Ion Proton platform.

### Sequence assembly

By base calling, the original image data produced by the sequencer is transferred into sequences, which are defined as “raw reads”. As there are some adaptor sequences and/or low-quality reads present in the raw reads, data filtering is carried out to obtain high quality reads as the clean reads. The procedure includes the following steps: 1. Remove reads when the length is less than threshold. 2. Trim reads adapter: if the length of the trimmed reads is less than the threshold, then remove the reads. 3. Calculate the average quality of 20 bases from the 3′ end until the average quality is larger than 9; then, trim the bases that have been counted. Clean reads are high quality sequences upon which all of the following analyses are based.Transcriptome de novo assembly is carried out with short reads assembling program–Trinity^32^. Trinity combines three independent software modules: Inchworm, Chrysalis, and Butterfly, applied sequentially to process large volumes of RNA-seq reads. The result sequences of trinity is called unigenes. When multiple samples from a same species are sequenced, unigenes from each sample’s assembly can be taken into further process of sequence splicing and redundancy removing with sequence clustering software to acquire non-redundant unigenes as long as possible.

### Gene identities assigned

The genes are firstly aligned by blastx (E-value < 0.00001) to protein databases in the priority order of NR, Swiss-Prot, KEGG and COG. That is, we first align Unigenes to NR, then Swiss-prot, then KEGG, and finally COG. The genes aligned to a higher priority database will not be aligned to lower priority database. The alignments end when all alignments are finished. Proteins with highest ranks in blast results are taken to decide the coding region sequences of the genes, and then the coding region sequences are translated into amino sequences with the standard codon table. So both the nucleotide sequences (5′-3′) and amino sequences of the gene coding region are acquired. The genes that cannot be aligned to any database are scanned by ESTScan, producing nucleotide sequence (5′-3′) direction and amino sequence of the predicted coding region.

### Screening of differentially expressed genes

We developed a strict algorithm to identify differentially expressed genes between two samples^33^. The total clean tag number of sample 1 is N1, and total clean tag number of sample 2 is N2. Gene A holds x tags in sample 1 and y tags in sample 2. The probability of gene A being expressed equally in the two samples can be calculated using the following equation:


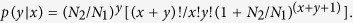


The P-value corresponds to the differential gene expression test. The FDR (False Discovery Rate) is a method for determining the threshold P-value in multiple tests. We assumed that we selected R differentially expressed genes and that S of the R genes really showed differential expression and the other V genes were false positives. We decided that the error ratio “Q = V/R” must remain below a cutoff (e.g., 1%); therefore, we set the FDR to a number that is not greater than 0.01^34^. We used FDR <0.001 and the absolute value of log2Ratio ≥1 as the threshold to judge significance differences in gene expression.

### Gene ontology analysis of DEGs

With Nr annotation, we used the Blast2GO program to obtain GO annotations of the DEGs. After obtaining GO annotations for the DEGs, we used WEGO software^35^ for GO functional classification of the DEGs and to understand the distribution of the gene functions of *M. alternatus* at the macro level. Furthermore, GO enrichment analysis maps all DEGs to GO terms in the database (http://www.geneontology.org/), calculates gene numbers for every term, and then uses hypergeometric tests to find significantly enriched GO terms in DEGs by comparison to the genome background. The formula used is:


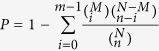


where N is the number of all genes with GO annotation; n is the number of DEGs in N; M is the number of all genes that are annotated to certain GO terms; m is the number of DEGs. The calculated p-value undergoes Bonferroni correction using a corrected p-value of ≤0.05 as a threshold. GO terms fulfilling this condition are defined as significantly enriched GO terms in DEGs.

### KEGG pathway analysis of DEGs

The formula used in the KEGG pathway enrichment analysis is the same as that used in the GO analysis. Here, N is the number of all genes with KEGG annotation, n is the number of DEGs in N, M is the number of all genes annotated to specific pathways, and m is number of DEGs in *M. alternatus.* Pathways with a P value < 0.01 are considered significantly enriched.

### Verification of Ion Proton data with RT-qPCR

We verified the Ion Proton data by RT-qPCR using the ΔΔCT method. Cytoplasmic actin of *M. alternatus* was used as the endogenous control and was normalized to be of equal quantity in each cDNA treatment. The primers for the RT-qPCR target genes were designed online using GenScript Real-time PCR Primer Design (https://www.genscript.com/ssl-bin/app/primer). The melting curves for each template/primer pair were examined for non-specific amplification. The RT-qPCR data were acquired on a LightCycler Real-Time PCR instrument using SYBR Premix Ex Taq^TM^ (TaKaRa, Japan) and universal thermocycler conditions according to the manufacturer’s protocol (Roche Diagnostics). Briefly, the PCR assay was conducted in 20-μl final volumes with a PCR mix containing 2 μl of cDNA at a concentration of 4 ng/μl (i.e., a quantity of cDNA corresponding to 20 ng of total RNA), 10 μl of SYBR Premix Ex Taq™, 0.4 μl of PCR Forward Primer, 0.4 μl of PCR Reverse Primer, and 7.2 μl of dH_2_O. The amplification scheme was 95 °C for 30 s, 95 °C for 5 s, and 60 °C for 20 s. The dissociation curve method was applied according to the manufacturer’s protocol (60 °C to 95 °C) to ensure the presence of a single specific PCR product. The annealing temperature was 60 °C, and RT-qPCR was performed using 40 cycles. For each cDNA, three RT-qPCR reactions were performed, and standard curves were generated. The threshold cycle (CT) and relative expression levels were calculated using LightCycler480 1.5 software (Roche Diagnostics).

## Additional Information

**How to cite this article**: Lin, T. *et al.* Identification of differentially expressed genes in *Monochamus alternatus* digested with azadirachtin. *Sci. Rep.* **6**, 33484; doi: 10.1038/srep33484 (2016).

## Figures and Tables

**Table 1:** Significantly enriched GO terms in DEGs (with P-values greater than or equal to 0.05).

Category	Gene ontology term	Cluster frequency (%)	P-value
Cellular component	Chromosomal part	3.0	0.00079
Molecular function	Acyl-[acyl-carrier-protein] hydrolase activity	1	2.61e-09
	fatty acid synthase activity	1.2	5.36e-09
	Catalytic activity	63.7	1.05e-07
	hydrolase activity	28	2.04e-05
	helicase activity	1.6	0.00128
	adenyl ribonucleotide binding	12.3	0.03219
	adenyl nucleotide binding	12.3	0.03508
Biological process	DNA metabolic process	6.6	8.63e-07
	epithelial cell migration, open tracheal system	0.7	7.03e-05
	epithelium migration	0.7	7.03e-05
	open tracheal system development	2.6	7.71e-05
	cell adhesion	2.9	0.00185
	heart development	0.9	0.00514
	respiratory system development	3.2	0.00631
	circulatory system development	1	0.03396
	cardiovascular system development	1	0.03396
